# Challenges in Diagnosing Adolescent Goiter: A Case Report with Brief Literature Insights in Juvenile Desmoid-Type Fibromatosis of the Thyroid Gland

**DOI:** 10.3390/jcm14020610

**Published:** 2025-01-18

**Authors:** Giorgiana-Flavia Brad, Iulius Jugănaru, Delia-Maria Nicoară, Alexandra-Cristina Scutca, Meda-Ada Bugi, Raluca Asproniu, Alexandru-Daniel Chelu, Diana-Georgiana Basaca, Mărioara Corneanu, Otilia Mărginean

**Affiliations:** 1 Department XI Pediatrics, Discipline I Pediatrics, ‘Victor Babeș’ University of Medicine and Pharmacy of Timișoara, 300041 Timișoara, Romania; brad.giorgiana@umft.ro (G.-F.B.); nicoara.delia@umft.ro (D.-M.N.); scutca.alexandra@umft.ro (A.-C.S.); asproniu.raluca@umft.ro (R.A.); diana.basaca@umft.ro (D.-G.B.); marginean.otilia@umft.ro (O.M.); 2 Department of Pediatrics I, Children’s Emergency Hospital “Louis Țurcanu”, 300011 Timișoara, Romania; bugi.ada@umft.ro; 3 Research Center for Disturbances of Growth and Development in Children BELIVE, ‘Victor Babeş’ University of Medicine and Pharmacy of Timișoara, 300041 Timișoara, Romania; chelu.alexandru@umft.ro; 4 Ph.D. School Department, ‘Victor Babeș’ University of Medicine and Pharmacy of Timisoara, 300041 Timișoara, Romania; 5 Department II of Microscopic Morphology, Pathological Discipline, “Victor Babeș” University of Medicine and Pharmacy, 300041 Timișoara, Romania; corneanu.marioara@umft.ro; 6 Research Center ANAPATMOL, ‘Victor Babeș’ University of Medicine and Pharmacy of Timișoara, 300041 Timișoara, Romania; 7 “Pius Brinzeu” County Emergency Clinical Hospital, 300723 Timișoara, Romania

**Keywords:** goiter, thyroid, juvenile desmoid-type fibromatosis, adolescent

## Abstract

The prevalence of goiter, thyroid nodules, and thyroid cancers in the pediatric population has increased. In some rare cases, local conditions such as juvenile desmoid-type fibromatosis (JDTF) can mimic specific thyroid pathology, complicating the diagnostic process. A 17-year-old obese adolescent girl was admitted to the Endocrinology Department with progressive swelling on the left side of the neck, persisting for approximately one year, recently accompanied by dysphonia and inspiratory dyspnea, and ultimately diagnosed as a unilateral nodular goiter associated with compressive phenomena. Despite her euthyroid status, the thyroid ultrasound identified a suspected, large, non-homogeneous, hypoechogenic nodule with calcifications in the left thyroid lobe (TI-RADS score of 4), confirmed by a cervical-region MRI. The biopsy specimens obtained through fine-needle aspiration were classified as Bethesda III (“atypia of undetermined significance” or “follicular lesion of undetermined significance”). Left thyroid lobe removal was performed by a specialized surgeon in thyroid pathology, with histopathological analysis revealing a diagnosis of JDTF in the thyroid gland. Post-surgery, the patient showed favorable progress without any relapse. Pediatric endocrinologists face challenges in diagnosing and managing thyroid nodules in children due to their higher malignancy potential. Familiarity with similar conditions, such as JDTF, is crucial in accurate diagnosis and appropriate pediatric management.

## 1. Introduction

Goiter, the most common indicator of thyroid disease in children and adolescents, represents the enlargement of the thyroid gland, where its volume exceeds two standard deviation scores for age and sex [1]. It may manifest regardless of specific thyroid hormone deficiency or excess symptoms.

Several factors can trigger the enlargement of the thyroid gland. Stimulation factors include elevated levels of thyroid-stimulating hormone (TSH), activated antibodies binding to the TSH receptor, or the activation of the G-protein (GNAS1), particularly notable in McCune–Albright syndrome [2]. Inflammation, whether acute infectious or non-infectious, such as subacute thyroiditis, can also contribute to goiter. Infiltration by malignancy, histiocytosis, lymphocytic thyroiditis, and tuberculosis is another pathway to thyroid enlargement [3].

Goiter manifests in two primary forms: diffuse, involving the enlargement of the entire thyroid gland, and nodular, characterized by solitary or multiple nodules on the thyroid gland. The incidence of thyroid nodules and thyroid cancers in the pediatric population is increasing due to the widespread use of thyroid ultrasound in the surveillance and diagnosis of thyroid conditions [4,5]. The estimated prevalence of thyroid nodules in children ranges between 1% and 1.7% [5], with a higher incidence observed in adolescents, where the rates increase to 13%, particularly when compared to younger children [4]. The prevalence ratio is 5:1 in favor of females [5]. Within the pediatric age group, thyroid nodules carry a significantly increased risk of malignancy, ranging between 22% and 25%, in contrast to adult patients (5–10%) [4,6].

In cases where ultrasound reveals a high suspicion of malignancy in a thyroid nodule characterized by a solid hypoechoic or solid hypoechoic component of a cystic nodule with irregular margins, microcalcification, taller-than-wide, or calcification, fine-needle aspiration (FNA) is recommended [7]. This is particularly crucial in children due to the increased rate of malignancy.

We aimed to illustrate the challenges encountered in diagnosing a 17-year-old adolescent girl admitted to the Endocrinology Department with goiter and compressive symptoms. The goal was to highlight the decision-making process for diagnosing and treating a patient with unilateral nodular goiter. Additionally, a brief literature review was conducted concerning juvenile desmoid-type fibromatosis (JDTF) of the thyroid gland, which was identified as the underlying cause of unilateral goiter in the presented case.

Following the Declaration of Helsinki, the patient’s parents provided informed consent to publish anonymized information in this case report. Furthermore, approval for the publication and dissemination of this manuscript was obtained from the Hospital’s Ethical Committee, ensuring compliance with ethical guidelines and protecting patient confidentiality (approval number: 1804/1 February 2024).

## 2. Case Report

### 2.1. Patient Information

We present the case of a 17-year-old Caucasian adolescent girl admitted to the Endocrinology Department of tertiary hospital “Louis Turcanu” Children’s Emergency Hospital, from Timisoara, Romania, for progressive swelling on the left side of the neck, persisting for approximately one year before hospitalization. Recently, she experienced symptoms such as dysphonia, inspiratory dyspnea, and 10 kg weight gain over the last 12 months.

The patient’s history showed her to be the only child of healthy, non-consanguine parents. She was born at term with an appropriate weight and height for her gestational age. There is no significant medical, traumatic, or familial history, including any thyroid disease or cancer.

### 2.2. Symptoms and Clinical Findings

The clinical examination revealed various pathological findings, including generalized well-distributed subcutaneous cellular tissue and evident abdominal obesity, reflected in the abdominal circumference of 114 cm (with a normal value for age and sex being 80.1 ± 1.61 cm) [8]. A globular chest was noted, along with white stretch marks in the lumbar region, arms, and thighs. The auxological parameters of the patient indicated a normal height for her age, increased weight (93 kg), and a BMI of 35 kg/m^2^, exceeding the 97th percentile for age and sex.

Examination of the anterior cervical region revealed a 5 × 3 cm soft, irregular, non-tender mass with ill-defined margins on the left side of the neck. The mass moved with swallowing and was localized at the thyroid lodge without any associated cervical adenopathy. The cervical perimeter (36 cm) was increased compared to the normal value for her age and sex [9,10]. No symptoms of pain, loss of appetite, swallowing, or mastication disturbance, or symptoms suggestive of thyroid pathology, were reported, and carotid pulsation was palpable.

Based on the clinical examination, the admission diagnosis was a unilateral nodular goiter with compressive symptoms and severe obesity.

### 2.3. Biological and Paraclinical Assessment

The patient was biochemically euthyroid (FT3 = 5.91 pmol/L, FT4 = 14.92 pmol/L, TSH = 1.73 µUI/mL), and the antibodies against the thyroid gland (thyroid peroxidase antibody = 12.2 UI/mL, thyroglobulin antibody < 15 UI/mL, TSH receptor antibody < 0.300 UI/L) were negative. All biological investigations were within the range, including the lipid profile and prolactin level. We will not detail the biological assessments and consultations conducted to evaluate obesity and complications unrelated to the presented pathology.

The ENT examination indicated that the dysphonia was secondary to a transient dysfunction in vocal cord motility caused by gastroesophageal reflux disease, with all other examinations yielding normal results.

**Figure 1:**
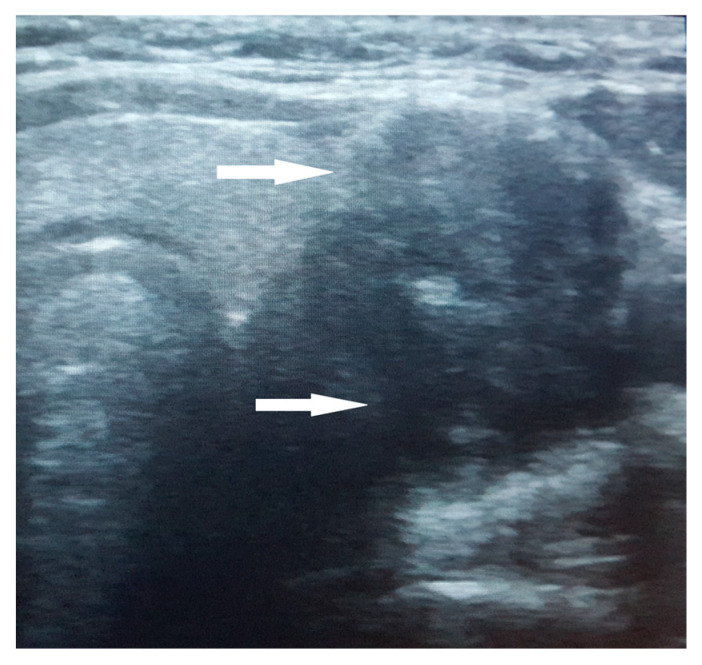
Ultrasound aspect of the large nodule in the left thyroid lobe. The nodule exhibits a non-homogeneous, hypoechogenic structure with poorly defined margins and internal calcifications (arrow).

The thyroid ultrasound revealed a notably large nodule within the left thyroid lobe, characterized by a non-homogeneous, hypoechogenic structure, poorly defined margins, and internal calcifications. A negative Doppler signal was described at the center of the nodule, while its periphery showed partial vascularization (Figure 1). No satellite adenopathy was detected. The total thyroid volume exceeded the normal range for her age and sex, measuring 17.7 mL. Strain elastography assigned a score of 5 (blue color), indicating hardness in the nodule region, while the TI-RADS (Thyroid Imaging Reporting and Data Systems) score was 4.

Given the unique characteristics and uncertain localization of this structure described in the ultrasound, discerning whether it belonged to the thyroid or parathyroid gland represented a challenge. Thus, more specific biological and imagistic investigations were conducted to address this.

Notably, the results for all thyroid tumor markers (calcitonin, carcinoembryonic antigen, thyroglobulin) came back negative.

**Figure 2:**
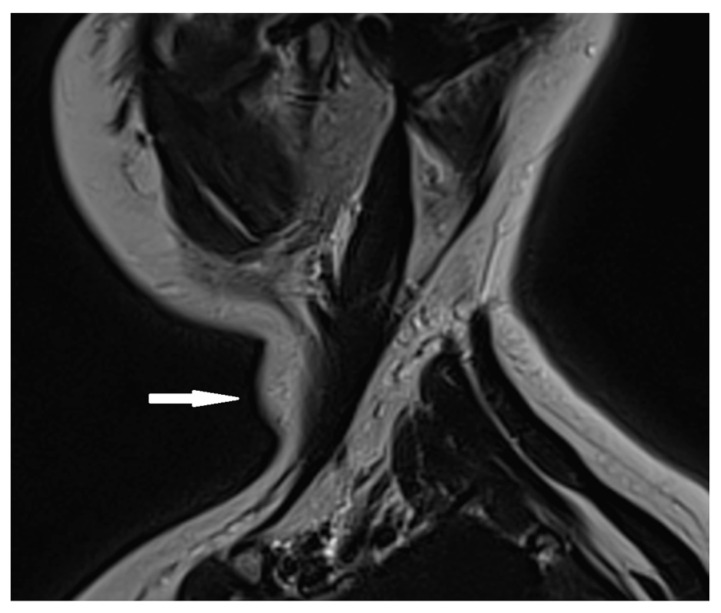
Cervical MRI with gadolinium contrast showing a gadolinium-avid tumor (arrow) located posteriorly to the left thyroid lobe.

Based on the ultrasound results, a cervical MRI with a contrast substance was conducted, revealing the presence of a gadolinium-avid tumor situated posteriorly to the left thyroid lobe. This tumor exerted pressure on adjacent structures, including the esophagus and trachea (Figure 2).

The exclusion of a parathyroid adenoma diagnosis was based on the absence of specific symptoms associated with hyperparathyroidism, such as bone pain, kidney stones, polyuria, constipation, or fatigue. Additionally, normal serum levels of parathyroid hormone, 25-hydroxy vitamin D, and phosphocalcic metabolism parameters (including calcium, phosphate, magnesium, and alkaline phosphatase levels) and the normal appearance of parathyroid glands were observed without any abnormalities described on the MRI.

In this situation, a fine-needle aspiration (FNA) of the thyroid nodule was performed, and biopsy specimens were collected from various nodule regions. The interpretation of the FNA sample was conducted by an experienced cytopathologist using the Bethesda System for Reporting Thyroid Cytopathology, resulting in the classification of the specimen as “atypia of undetermined significance” or “follicular lesion of undetermined significance” (Bethesda III).

### 2.4. Therapeutic Intervention

According to the American Thyroid Association Guidelines [7], the tumor surgery was performed by an experienced surgeon specializing in thyroid pathologies.

**Figure 3:**
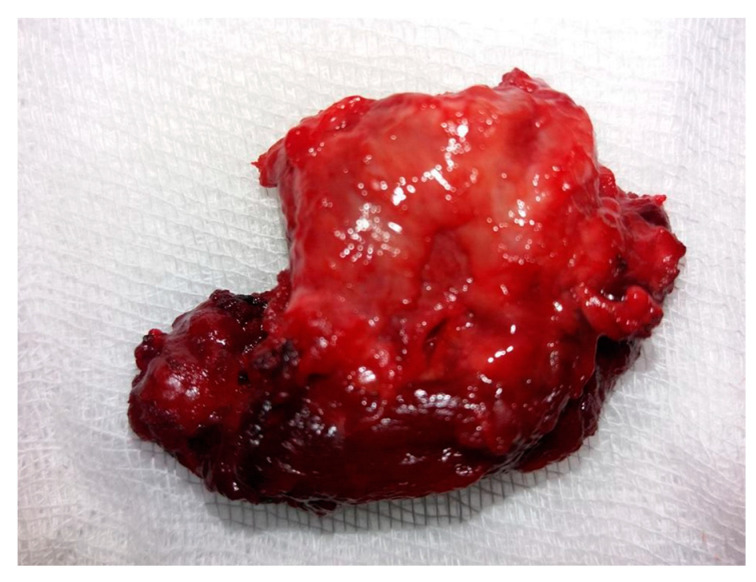
Macroscopic aspect of the tumor: a 1.8 × 1.6 × 1.3 cm specimen characterized by a non-uniform aspect, creamy coffee color, and brown stripes.

A tumor exhibiting increased consistency was surgically removed, along with the entire thyroid lobe. The mass was firmly adhered to the adjacent tissues. Sectioning the isthmus and separating the left thyroid lobe from the trachea and thyroid cartilage proved challenging. Consequently, a left lobectomy was performed with adequate local hemostasis. No evidence of adenopathy was observed in the left lateral cervical compartment. On macroscopic histopathological examination, the thyroid specimen measured 4.1 × 2.6 × 1.2 cm and exhibited a brown-pink color with an irregular shape, elastic consistency, and a smooth surface upon palpation. The tumor (Figure 3) was distorted and contained in the left lobectomy specimen, situated in the lower external third, with dimensions of 1.8 × 1.6 × 1.3 cm. It displayed a brown-mahogany coloration reminiscent of coffee, an irregular shape, and a firm consistency compared to the surrounding normal thyroid tissue. Superficially, the tumor exhibited multiple irregular peripheral brown streaks, creating a striped appearance that converged and aligned with the adjacent thyroid tissue.

Five longitudinal sections were made along the right axis of the tumor and adjacent left thyroid tissue. The normal thyroid tissue displayed a preserved architecture with a non-homogeneous appearance. The tumor section exhibited a fleshy, inhomogeneous, non-uniform texture and a mahogany-brown color. Multiple abundant spirals of breezes were observed at the periphery of the tumor, blending with the adjacent normal thyroid tissue and showing an “extension by grasping” pattern. This aspect was less prominent in the central areas of the tumor. Serial incisions were performed to evaluate the tumor, its borders, and the adjacent supposedly normal thyroid tissue.

**Figure 4:**
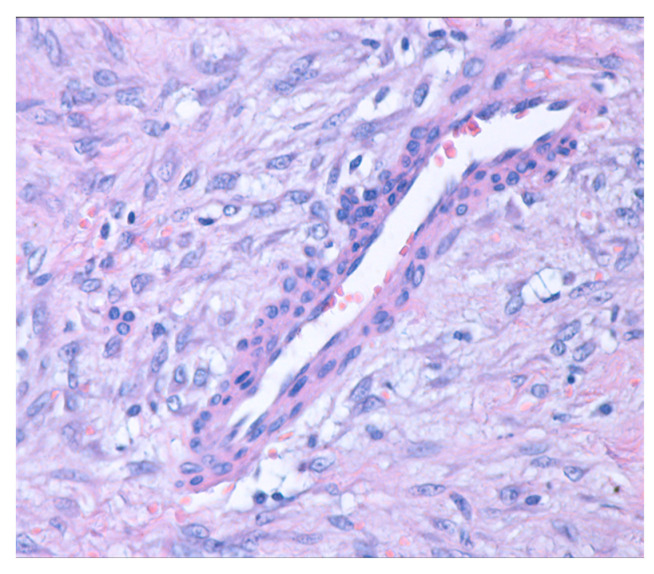
Mesenchymal proliferation with nodular growth and infiltrative edges (hematoxylin and eosin stain; 40× magnification).

On histopathological examination, on hematoxylin and eosin staining, the thyroid parenchyma showed multiple typical medium-sized thyroid follicles, occasionally forming nodules and fibrinous bands. These were separated by fibrous and vascularized interstitial tissue, with inclusions of small foci of apocrine metaplasia. Additionally, a tumoral mesenchymal proliferation (Figure 4) was observed. This proliferation was well demarcated, exhibited nodular growth, and had infiltrative margins into the residual thyroid, which appeared to be within normal histological limits.

A few focal ectatic, thin- and thick-walled blood vessels were interspersed within the tissue, but no evidence of malignancy or obliterative vasculitis was observed. Examination of the tumor revealed a bland appearance with relatively uniform, low-grade, fusiform, well-differentiated intermediate collagen-laid fibroblastic tumor cells with abundant pale cytoplasm and fusiform–oval core, separated from the blood vessels and set in a collagenous matrix. The core occasionally shows a nucleolus without atypia or pleomorphism. A minimal focal inflammatory infiltrate was noted within and at the leading edge of the lesion, composed of mast cells, lymphocytes, plasma cells, and rare macrophages.

**Figure 5:**
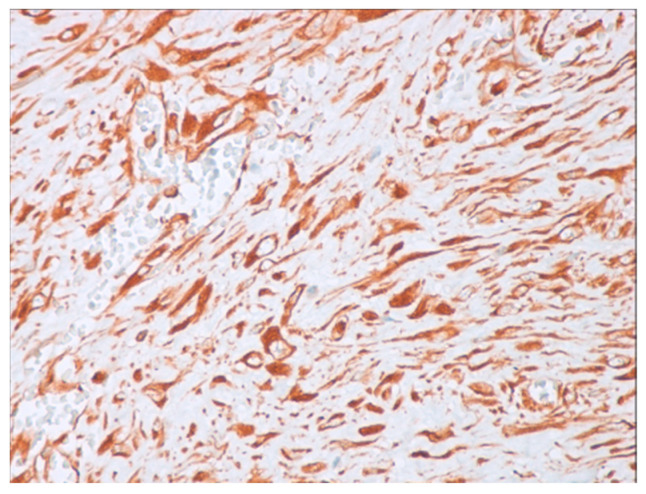
Positive immunohistochemistry for vimentin (40× magnification).

**Figure 6:**
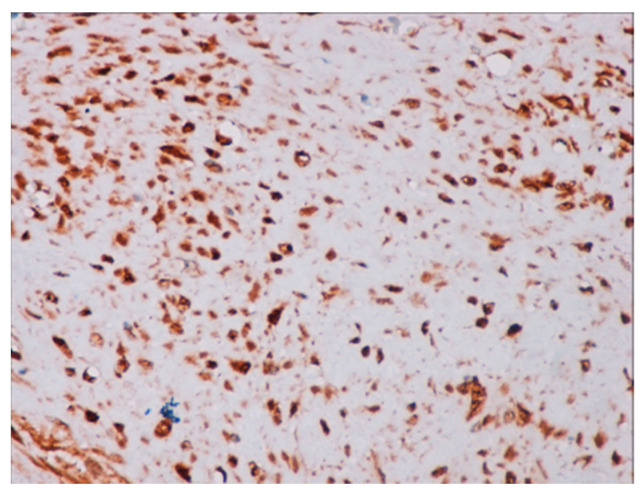
Positive immunochemistry for β-catenin with a strong nuclear pattern (40× magnification).

**Figure 7:**
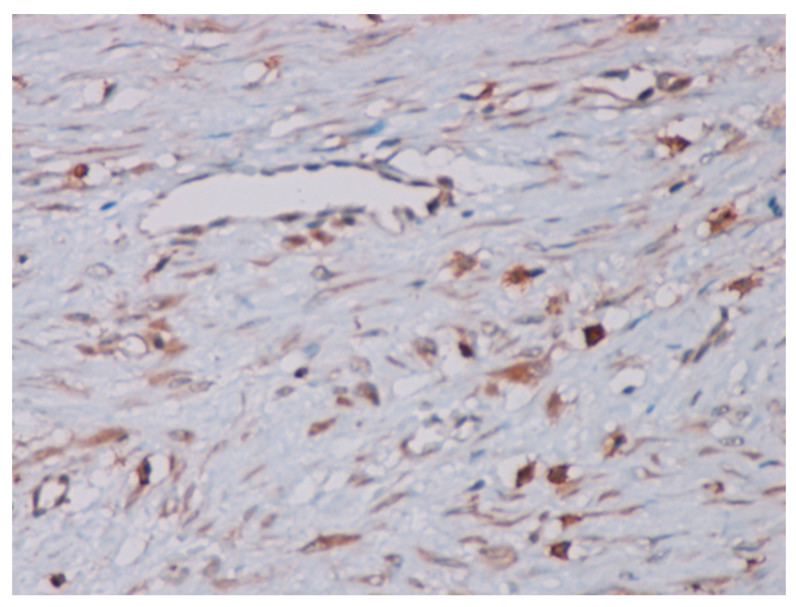
The mast cells positive for the C-Kit dispersed along blood vessels (40× magnification).

Immunohistochemical studies demonstrated that the cells showed zonal expression for smooth muscle actin (BD Biosciences (Franklin Lakes, NJ, USA)), desmin (BD Biosciences, clone D33, 610711), and vimentin (BD Biosciences, clone V9, 550513) (Figure 5), and stained nuclear and cytoplasmic with β-catenin (BD Biosciences, clone 14, 610153) (Figure 6), but were negative for pan-cytokeratin, CD34 and S-100. The tumor proliferation index Ki-67 (BD Biosciences, clone B56, 556003) was 5% in the areas with the highest proliferation. Mast cells are positive for C-Kit and were distributed along the blood vessels, while the inflammatory infiltration was located between the lesion and the surrounding adjacent soft tissues (Figure 7).

Histopathological findings and immunohistochemical results correlated with the clinical features of slow tumor growth dynamics support the diagnosis of juvenile desmoid-type fibromatosis (JDTF) with a conventional pattern (ICD-O: 8821/1) and focal involvement of the thyroid gland.

### 2.5. Follow-Up

After the left lobectomy, the patient developed dysphonia and dyspnea due to transitory paralysis of the left vocal cords for almost three months, with spontaneous remissions.

During post-surgical follow-up to monitor for recurrence, the patient remained asymptomatic, showed significant improvement in overall well-being, gained weight, and experienced no relapse in the cervical region. Normal thyroid function was maintained (TSH within normal range and serum thyroglobulin level below 2.0 ng/mL) despite the absence of hormonal replacement therapy secondary to the left thyroid lobe removal.

An evaluation of the CTNNB1 mutation status could not be performed, limiting our ability to investigate its potential role in this case and to exclude any associated syndromic conditions.

Notably, there was no suggestion of clinical or imagistic cervical tumoral relapse throughout the monitoring period. The absence of recurrence was consistently confirmed through multiple ultrasounds and periodic neck CTs. Assiduous monitoring and follow-up efforts have been diligently sustained up to the present, extending over 35 months. This continuous and meticulous supervision further reinforced the confirmation of a sustained positive outcome in the patient’s post-surgical journey, culminating in the birth of a baby almost two years after the surgery.

## 3. Methods

This narrative review was conducted using the PubMed search engine to identify relevant studies published between 1 January 2000, and 30 November 2024. The search employed the following keywords, used individually or in combination: “goiter”, “thyroid”, “juvenile”, “desmoid-type fibromatosis”, “adolescent”, and “pediatric”. Only articles published in English were considered. Eligible publications included case reports, case series, studies, and review articles. The review focused on several key domains: the definition, epidemiology, clinical manifestations, histopathological features, pathology, and treatment of JDTF.

## 4. Discussion

### 4.1. Definition and Epidemiology

According to the World Health Organization, desmoid-type fibromatosis (DTF) is classified as a borderline soft tissue tumor with mesenchymal origin. This rare entity involves the monoclonal proliferation of fibroblasts in muscles, ligaments, and tendons, exhibiting intermediate biological behavior with low malignant potential. They are characterized by slow growth, infiltrative growth patterns, and a tendency toward local aggressiveness and recurrence, without the ability to metastasize [11]. Despite being prone to frequent relapses (24–76%) and episodes of progression, DTF notably lacks the ability for distant spread, with 10-year overall survival higher than 90% [12,13]. Some DTFs can be life-threatening due to their location (cervical and profound abdomen occurrences).

DTF accounts for approximately 0.03% of all neoplasms and represents less than 3% of all soft tissue tumors [14,15]. Its annual incidence is relatively low, with 3–4 cases per 1 million individuals, and it has a female-to-male ratio of 3:1 [16]. Two incidence peaks were described: one during childhood (ages 6–15 years) and another in women from puberty to 40 years old.

Based on the age group that it predominantly affects, DTF has two forms: juvenile (JDTF) and adult variants. Rapid growth and recurrence are most commonly associated with the juvenile forms of the disease. It may present as a single lesion or multiple lesions, with the potential for widespread distribution.

The DTF location is classified as abdominal (6–8% of cases) and extra-abdominal. A quarter of all DTFs occur in children under 15 years, particularly in boys. Children are more frequently affected by head and neck JDTF (26–33% of cases) than adults (only 7–9% of cases), making it the second most common site after localization in the trunk and limbs [17,18]. The most commonly affected sites in the head and neck region are the tongue, mandible, and mastoid processes, while the involvement of the thyroid, hypopharynx, and larynx is rare [19,20].

The case presented illustrates a rare instance of JDTF of the thyroid gland in a 17-year-old adolescent girl. The tumor was adherent to the trachea and major neck vessels. Its increased consistency caused the displacement of the left thyroid lobe, creating a false impression of a thyroid nodule and presenting a diagnostic challenge for both the endocrinologist and the pathologist.

A few articles report cases of the DTFs of the thyroid gland [19,21,22,23]. These cases involved patients aged 26 to 68 years, mostly female, commonly presented with goiter and symptoms of organ compression. Some cases were associated with concurrent papillary thyroid carcinoma or were diagnosed after thyroid cancer surgery. All patients underwent surgery, but nearly half experienced recurrences that required additional treatments for disease control.

### 4.2. Clinical Manifestations

The presentation of JDTF ranges from asymptomatic, firm, slow-growing tumors to those that invade nearby structures. It typically has a favorable prognosis and is usually limited to the musculoskeletal system [17,24,25].

Clinically, these tumors are typically painless masses detectable by palpation or imaging and can occur anywhere in the body. Their manifestations depend on the location, tumor size, and rate of progression, potentially exerting a severe impact on vital organs, causing functional limitations, and leading to life-threatening conditions [25,26,27].

The JDTF of the thyroid gland becomes symptomatic as it grows. Symptoms are often nonspecific and may mimic common childhood illnesses. These can include cough, dyspnea, and pain due to the mass effect. However, infiltration into the cervical organs can result in life-threatening complications.

Our patient first detected the tumor at the age of 16 and noticed its progressive growth. Due to its location in the anterior cervical region, at the posterior part of the left thyroid lobe, and its firm characteristics, the JDTF mass, along with the goiter, exerted pressure on the larynx and trachea, causing symptoms such as dyspnea and inspiratory difficulty. The patient consulted an endocrine specialist only after complications had developed. The presence of these symptoms imposed a thyroid ultrasound, which identified a “suspected thyroid nodule” with a TI-RADS score of 4. Following pediatric guidelines, FNA was performed, and biopsy specimens were collected [7,28]. The FNA sample was interpreted as Bethesda category III, classified as “atypia of undetermined significance” or “follicular lesion of undetermined significance”. A lobectomy was performed based on the 2015 American Thyroid Association Guidelines (ATA) Guidelines [7]. Pathological examination confirmed the diagnosis of JDTF with a conventional pattern and focal involvement of the thyroid gland. The tumor’s adherence to adjacent organs and large neck vessels suggests a life-threatening risk in the absence of surgery. Fortunately, the outcome was favorable and the patient did not experience any recurrence post-operatively.

More recently, the 2022 European Thyroid Association (ETA) guidelines introduced a revised approach to Bethesda III nodules, recommending repeated FNA after six months and the consideration of genetic testing to detect oncogenic drivers or gene fusions (e.g., RET/PTC and NTRK fusions) [28]. This update aims to refine diagnosis and minimize unnecessary surgical interventions. A question arises: what would have happened to the tumor if FNA had been repeated after six months, as recommended by the 2022 ETA guidelines, given its adherence to adjacent organs and major neck vessels?

The 2015 ATA Guidelines and the 2022 ETA Guidelines both address pediatric thyroid nodules and thyroid cancer, with papillary thyroid carcinoma being the most common malignancy in this population. Both emphasize the need for pediatric-specific approaches, recognizing the distinct differences in disease presentation, prognosis, and treatment response compared to adults. These guidelines aim to deliver safe and effective care for pediatric patients with thyroid cancer [7,28]. The 2015 ATA Guidelines serve as a foundational reference and continue to be widely utilized, whereas the 2022 ETA Guidelines incorporate more recent data, prioritizing conservative management and reducing invasive procedures when appropriate.

Following the diagnostic pathways for thyroid nodules outlined in these guidelines (e.g., thyroid ultrasound, FNA, and histopathological examination), we ruled out malignancy and identified the tumor as a borderline condition, such as JDTF.

### 4.3. Histopathological Diagnosis

Histopathological examination is the key element of DTF, and therefore, it is crucial to pay special attention to this aspect of diagnosis. The proper interpretation of these histopathological findings is vital in confirming diagnoses and guiding treatment strategies.

DTF typically presents as a firm, encapsulated tumor with indistinct margins. Macroscopic features of DTF include the presence of mahogany-brown fibrin bands on the tumor surface and at the interface of the tumor and the adjacent supposedly healthy parenchyma. This interface often demonstrates a “grip extension” pattern, characterized by the formation of spoke-like membranes. Microscopic criteria on hematoxylin and eosin staining include, as distinctive features, a bland fibroblast/myofibroblast appearance characterized by well-differentiated fusiform cells, collagenous stroma, and bands of fibrous tissue interspersed with thin-walled blood vessels in the absence of significant nuclear atypia.

It exhibits seven distinct morphological patterns, the most common being the conventional type, hypoplastic/hyalinized, and staghorn vessel variants [29]. Our adolescent patient was diagnosed with the conventional JDTF type, which is considered a milder form.

The histopathological interpretation of DTF can be challenging due to overlapping histological features with other soft tissue tumors, highlighting the importance of immunohistochemical markers in accurate differentiation and diagnosis. They exhibit a unique immunohistochemical profile, marked by nuclear positivity for β-catenin, consistent positivity for vimentin, and smooth muscle actin [30]. The tumor cells uniformly lack the expression of desmin, S-100, and CD34. Histopathological differential diagnoses can be challenging, as DTF shares microscopic similarities with several entities, such as Gardner’s fibroma, nodular fasciitis, myofibromatosis, or fibrosarcoma.

Thus, the diagnosis of DTF requires expertise in mesenchymal tumors and must be confirmed by a skilled pathologist. This diagnosis should be correlated with clinical criteria, including the tumor’s slow progression and the absence of metastatic potential.

JDTF must be differentiated from other pediatric tumors occurring in the cervical region, including lymphomas, Langerhans cell histiocytosis, germ cell tumors, and soft tissue lesions such as rhabdomyosarcoma, neurogenic tumors, or primitive neuroectodermal tumors. It should also be distinguished from other thyroid gland diseases like Riedel’s thyroiditis or papillary thyroid carcinoma with fibromatosis-like stroma or nodular fasciitis-like stroma. These latter terms are synonymous with papillary thyroid carcinoma with desmoid-type fibromatosis and represent a distinct histological subtype of papillary thyroid carcinoma, not an association with a separate malignant thyroid tumor [31].

### 4.4. Pathology

The exact etiology of aggressive DTFs is unknown, but local trauma, hormonal fluctuations, and genetic conditions are involved.

Physical factors such as trauma (including surgical trauma) and radiation are considered to be responsible for the development of the tumor [30].

Estrogen levels have been associated with tumor progression, suggesting that it is based on hormones. This is supported by the higher incidence of tumors during and after pregnancy, as well as after exposure to oral contraceptives, and the spontaneous regression observed during menopause [32]. 

Most cases of DTFs (90%) arise sporadically due to somatic mutations, primarily in the *CTNNB1* gene, which encodes β-catenin [33]. These mutations typically affect codons 41 and 45 of exon 3 [34] and are more commonly observed in women [27]. In adults, *CTNNB1* mutations account for over 85% of cases, whereas in children, the mutation spectrum is more complex, with mutations in *AKT* (31%), *BRAF* (19%), and *p53* (9%) being more frequent [17,35,36]. The *CTNNB1* gene produces the β-catenin protein, while the *APC* gene encodes a protein that regulates β-catenin levels.

In hereditary forms of DTFs (5–10% of cases), a tumoral pathogenic variant in the *APC* gene is linked to conditions like familial adenomatous polyposis or Gardner’s syndrome [37,38,39,40] activating the canonical *Wnt/β-catenin* signaling pathway [34,38]. Individuals with these genetic pathologies have a significantly higher risk of developing DTFs—approximately 1000 times greater than the general population, with an estimated incidence of 5–16% for developing the condition [41].

Mutations in the *APC* and *CTNNB1* genes are mutually exclusive, meaning that DTFs with a mutation in one gene generally do not have mutations in the other [41]. This exclusivity helps in identifying syndromic conditions, such as *APC*-related syndromes, when *CTNNB1* mutations are absent. The biological assessment of *CTNNB1* mutation status in tumors is recommended to rule out other differential diagnoses and help exclude any associated syndromic conditions [42,43].

In the case presented, the adolescent patient is at an age characterized by elevated estrogen levels due to active puberty and reproductive system maturation. Also, it is well known that increased adipose tissue/obesity is associated with elevated estrogen levels, primarily due to the aromatization occurring within the adipose tissue [44]. This supports the conclusion that the tumor’s progression is hormonally driven. Furthermore, she has no history of trauma or a familial history of colonic polyps or colorectal cancer. Unfortunately, it was impossible to evaluate *CTNNB1* mutation status to exclude any associated syndromic conditions. Recommendations were made to perform periodic colonoscopies to identify and monitor the appearance of polyps.

### 4.5. Treatment

The National Comprehensive Cancer Network guidelines recommend various primary and recurrent DTF treatments, including surgery, radiotherapy, systemic therapy, and observation [45].

In managing JDTF in pediatric patients, treatment is guided by tumor progression, factors like local aggressiveness, symptoms, growth potential, recurrence risk, and impact on organ function. Early surgical intervention may reduce morbidity, particularly if the tumor poses a life-threatening risk [35]. The main factor in decision-making is the tumor’s effect on the patient’s quality of life. Favorable prognostic factors include a tumor size of less than 5 cm, a head and neck location, and an age under 10 years [35,46].

A conservative “*watch-and-wait*” approach was recommended by the European Pediatric Soft Tissue Sarcoma Study Group (EpSSG) as the first-line treatment for tumors that did not progress over a 2–3-year period, with regular clinical and radiologic follow-up to prevent unnecessary treatment [47].

Exceptions are made for tumors in critical areas, such as the head, neck, and intrathoracic regions, where complete surgical excision with clear margins is the primary treatment for symptomatic JDTF to prevent serious complications. The surgical challenge is greater in the cervical area due to vital structures requiring wide tumor resection. Surgery is also recommended for tumors larger than 5 cm or in younger patients (<10 years).

Historically, margin-free surgical resection has been the preferred treatment. Currently, it is advised not to pursue a margin-negative resection if it would lead to the loss of function or significant morbidity [48].

In our patient’s case, the tumor was surgically removed with clear margins, as described in the histopathological evaluation. Following extensive discussions, a decision was made to proceed with surveillance. Periodic MRI scans were conducted to monitor the surgical outcome. She experienced no recurrence in the cervical region over a 35-month follow-up period.

Local recurrence rates post-surgery remain high, especially in children, with a recurrence ranging from 24% to 76% [49,50] despite surgical resection being the primary treatment in 23% to 83% of cases, and often impact quality of life [51]. Spontaneous regression may occur, occasionally following an initial phase of tumor growth [52].

Adjuvant radiotherapy or chemotherapy is recommended based on the status of the surgical resection margins.

Chemotherapy is recommended for JDTF with rapidly growing or unresectable tumors or those experiencing symptoms like pain, breathing, or eating difficulties to reduce the tumor size for eventual resection, either as a standalone or combined therapy. For chemotherapy-resistant cases, alternative treatments are considered based on individual factors [53].

Radiotherapy is generally avoided in children due to potential adverse effects, as well as the significant long-term risk of functional sequelae and the increased likelihood of developing second malignancies, in addition to a certain degree of radioresistance [54].

In pediatric cases, experimental treatments for this tumor are being explored, including newer therapies such as tyrosine kinase inhibitors targeting vascular endothelial growth factor receptors (VEGFR 1, 2, and 3), platelet-derived growth factor receptors (PDGFR α and β), and c-Kit (e.g., Imatinib, Sorafenib, Sunitinib, Pazopanib) [55]. Additionally, anti-inflammatory agents like Meloxicam, Indomethacin, Sulindac, and Celecoxib, along with biological treatments, are emerging as potential future therapies [46,56,57,58]. Tamoxifen, an antiestrogen agent, has also been considered a promising treatment for JDTF, based on evidence suggesting the tumor’s dependence on sex hormones, observed during different hormonal states in adults, such as pregnancy, menopause, and the use of contraception.

There are limited data in the medical literature regarding the management of JDTF of the thyroid gland in children or adolescents, particularly in recurrent or aggressive cases where surgery alone is insufficient. In such cases, attention has been directed toward medical strategies used in adults, as recurrence is a possibility. Low doses of sorafenib, a tyrosine kinase inhibitor, have been successfully used in adults to treat these severe DTFs following surgery for co-existent papillary thyroid carcinoma as a distinct and separate tumor or in recurrent cases. The treatment was well tolerated and achieved effective disease control [19,21]. While sorafenib is recommended for papillary thyroid carcinoma in pediatric patients [59], no data currently exist on its effects in managing JDTF involving the thyroid gland.

## 5. Conclusions

We report a rare case of JDTF involving the thyroid gland in a 17-year-old adolescent girl. Few cases with similar pathology have been described in the literature, and this is the only one reported during adolescence. The patient presented with a unilateral nodular goiter and symptoms caused by tumor-related organ compression. Surgical resection with clear margins was performed, and no recurrence was observed after three years. Her post-operative course was favorable, and she successfully gave birth three years after the operation.

The management of JDTF of the thyroid gland poses significant challenges for pediatric endocrinologists due to its rarity, atypical localization, clinical presentation, and histological features, which can be easily misinterpreted. Although JDTF is classified as benign, its locally invasive behavior and histological similarity to malignant tumors complicate diagnosis and treatment. Histopathological evaluation plays a pivotal role in accurate diagnosis, as it differentiates JDTF from other thyroid and cervical pathologies, including malignancies. Comprehensive histopathological analysis informs individualized treatment strategies and ensures precise differentiation, crucial in guiding clinical decisions, mainly regarding surgical excisions with microscopically negative margins.

Despite successful surgery, the significant risk of local recurrence necessitates long-term surveillance for timely detection and management. Sustained monitoring and a multidisciplinary approach are essential in addressing recurrence risks and optimizing patient outcomes in pediatric endocrinology.

## Data Availability

The data are not publicly available for privacy reasons.
